# Influence of patellar implantation on the patellofemoral joint of an anatomic customised total knee replacement implant: A case study

**DOI:** 10.1177/0954411920941400

**Published:** 2020-07-29

**Authors:** Linjie Wang, Chang Jiang Wang

**Affiliations:** Department of Engineering and Design, University of Sussex, Brighton, UK

**Keywords:** Patellofemoral contact force, relative motions, customised total knee replacement, squatting motion, musculoskeletal finite element model

## Abstract

Few studies have been conducted to investigate kinematics and kinetics of the patellofemoral joint under physiological muscle forces and ankle joint loads. In this study, a preliminary design of a customised total knee implant was proposed and created. To compare the influences of different patella treatment scenarios, a dynamic knee simulation model was created with patient-specific muscle forces and ankle joint loads that are calculated from an OpenSim musculoskeletal model. The goal is to improve patellar implant-bone connection and restore patellofemoral joint mobility. Identical dynamic boundary conditions were applied on an unresurfaced patella and three different dome-shaped patellar implants. It was found that the unresurfaced patella and patellar implants resulted in different motions of patellar internal rotation and medial tilt. The size of the dome-shaped patellar implant affected the motion and loading of the patellofemoral joint. When the exposed patella bone was not fully covered by the patellar implant, the patella bone then contacted the femoral component during knee flexion. This would most likely lead to anterior knee pain and subsequent revision.

## Introduction

Patellar resurfacing during total knee arthroplasty (TKA) remains controversial. It is usually performed in the presence of anterior knee pain, inflammatory arthritis, patellar mal-tracking and damaged articular cartilage. Many surgeons resurface the patella to avoid the patient developing postoperative anterior knee pain and the need for revision surgery.^1^

To study the influence of resurfaced and unresurfaced patellae on the motions and loading of the patellofemoral joint, methods such as in-vitro experiments^2–5^ and computational simulations^6–9^ have usually been implemented. Experimental methods based on the Oxford knee rig (OKR)^10,11^ and its derivative simulations^8,9^ can help demonstrate the patellofemoral relative motions but without any consideration of the practical ground reaction forces and complicated muscle coordinated effects. However, too much simplifying of the muscle forces and external forces on the lower limb may make prediction of the patellofemoral loads less accurate. Browne et al.^4^ tested central dome-shaped and medialized patellar implants on two different femoral components placed on six human cadaver knees based on the OKR. No significant differences in patellofemoral compressive and shear forces were observed between the two patellar implant designs. Baldwin et al.^6^ created a finite element (FE) knee simulation model based on the Kansas Knee Simulator^12,13^ to study patellofemoral and tibiofemoral joint motions. The simulation was similar to that of the OKR but included ankle joint medial–lateral force, internal–external and flexion–extension torques. Fitzpatrick and Rullkoetter^7^ studied the patellofemoral joint motions and contact stresses of three different commercial implants using fluoroscopy tibiofemoral kinematics to drive an FE model and applying a 1000-N ramped load on the quadriceps muscle.

There have also been some musculoskeletal simulations based on experimental motion capture data and inverse dynamic algorithms^14–16^ to calculate patellofemoral loads. However, normally those simulations overlook the practical complexity of patellar motion by being simplified to a single fixed patellofemoral flexion–extension axis and a pre-defined circular patellar trajectory. This may also make the prediction of both patellofemoral load and motion less accurate.

In this study, a dynamic FE knee simulation model was used to investigate the influence of patellar implantation on the patellofemoral joint forces and relative motions of a customised total knee implant (CTKI) that was anatomically designed. Different from the OKR experimental load conditions, the knee simulation model in this study used patient-specific muscle forces and ankle joint reaction loads that had been calculated using OpenSim with consideration for the effect of ground reaction forces during the squatting motion. The muscle and ankle joint loads were obtained by satisfying evaluation threshold of residual force in OpenSim RRA simulation. Different also from the pre-defined simplified kinematic relationship of the patellofemoral joint in the musculoskeletal models of previous studies, the patellofemoral motion in this study was affected and determined by the patellofemoral articulation contact shapes and loads the joint was subject to. The pre-strains in the patellofemoral collateral ligaments (PFCLs) in the unresurfaced patellofemoral joint were varied to investigate the influence of laxity of the PFCLs on the simulated results. Three different sized dome-shaped patellar implants were modelled to investigate patellofemoral joint dynamic responses and contact stresses. The simulated patellofemoral contact forces and relative motions were compared with published results from both experimental measurements and simulations.

## Material and methods

### Model of CTKI

**Figure 1.**
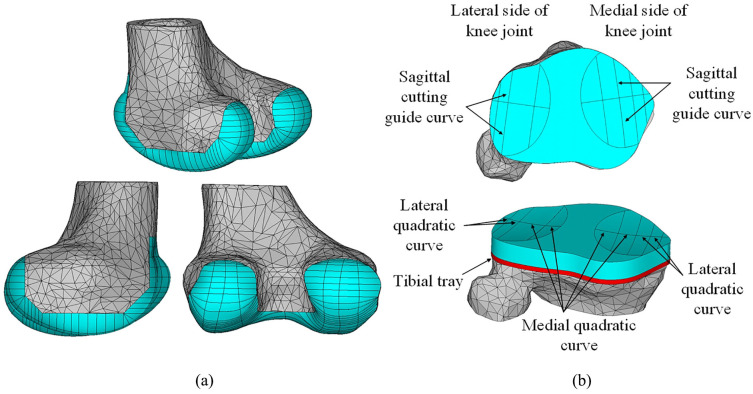
Customised total knee implant (CTKI): (a) femoral component and (b) tibial insert.

The same CTKI model which was developed in a previous study^17^ on CTKI design and its dynamic performance assessment was used in the current study. The femoral component (Figure 1(a)) was based on the left knee anatomy of subject JW for the 4th Grand Challenge Competition to Predict In Vivo Knee Loads^18^ (available from https://simtk.org/projects/kneeloads). The tibial bearing surface (Figure 1(b)) was based on the shape of the femoral condyles by defining an elliptical cutting guide track in the longitudinal direction and two quadratic curves in the transverse direction on each condyle.

### Geometries of the unresurfaced and resurfaced patellae

**Figure 2.**
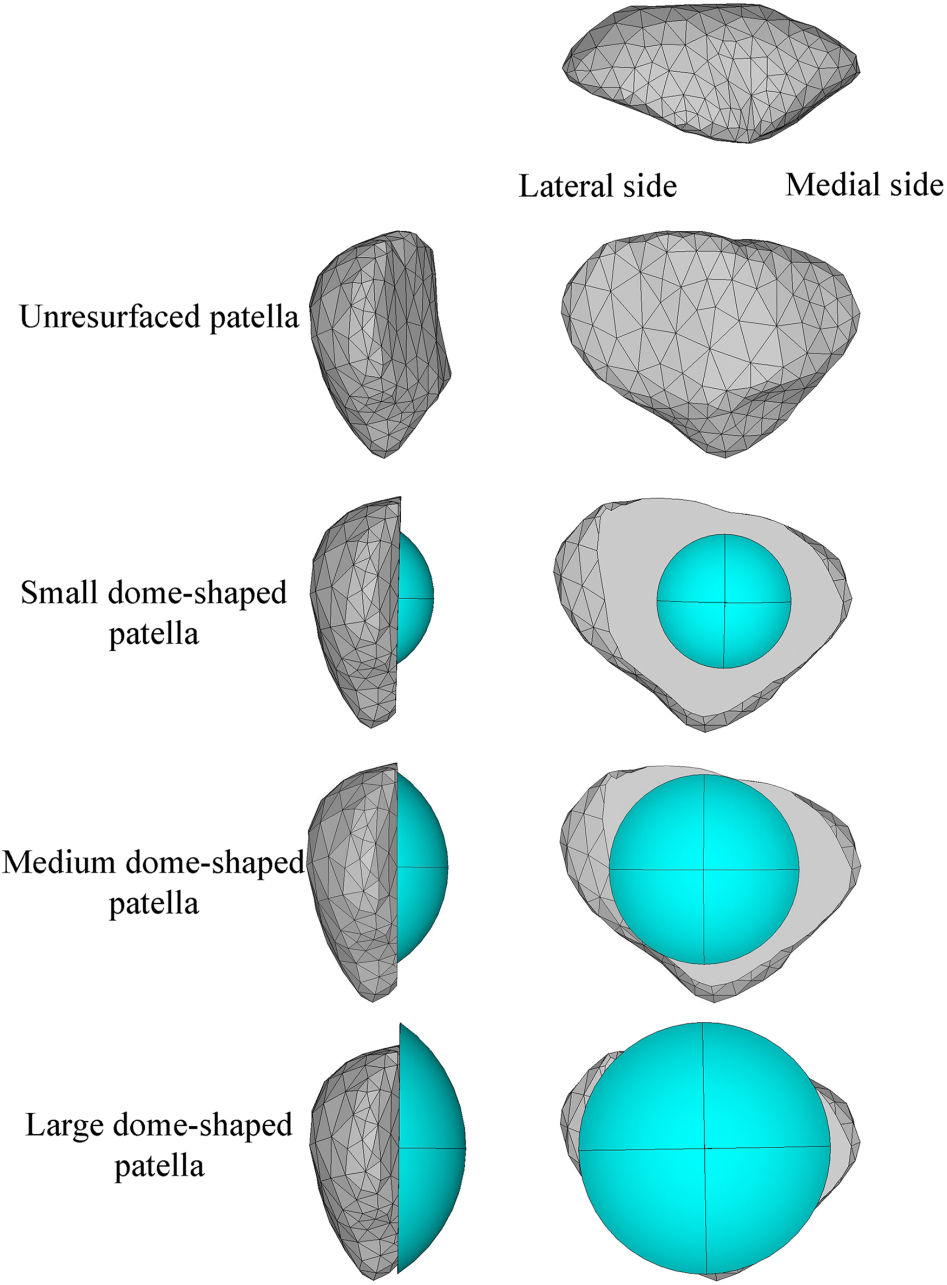
Models of unresurfaced patella and three different sized dome-shaped patellar implants.

The unresurfaced patella model was created from the CT images of the patella of subject JW. The articulation surface of the unresurfaced patella was removed for installing the patellar implant. Three different sized dome-shaped/button components were modelled and are shown in Figure 2. After determining the centre of the resected surface, a small dome-shaped patellar implant was created (third row in Figure 2). The size of this implant was referenced from the literature,^19^ with a base radius of 15 mm and a depth of 8 mm. This was regarded as an extreme condition for this study, as the patellar implant of this size would not be used by surgeons in normal circumstances. It is only analysed in this study for comparison with other patellar implants. The small implant was then scaled up until it was aligned with the patellar superior edge giving a medium-sized implant (fourth row in Figure 2). A large implant (fifth row of Figure 2) was modelled by scaling the initial implant further until the resected patellar bone was completely covered. The centres of the medium and the large implants were adjusted to reach the top and side of the resected surface.

### Dynamic knee simulation FE model

**Figure 3.**
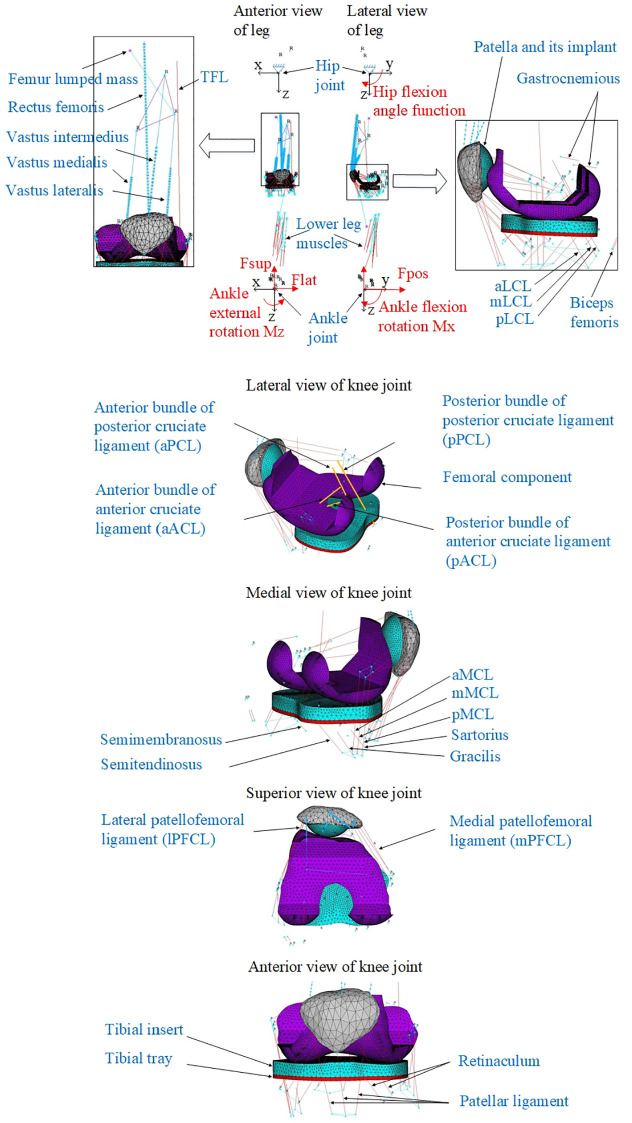
Dynamic knee simulation finite element model.

To assess the dynamic responses of the total knee implants, a dynamic FE knee simulation model was created with reference to the OKR. It has been successfully used to investigate the influence of three different cruciate ligament treatment scenarios on the tibiofemoral motions and compressive forces.^17^ As shown in Figure 3, the hip and ankle joints were created using the ANSYS multipoint constraint joint elements (MPC184) based on the locations of the hip and ankle joints centres of subject JW relative to the knee joint centre. Due to the lack of control mechanism in the current dynamic FE analysis, the hip joint was specified to have only 2 rotational degrees of freedom (DOFs) that represent flexion–extension, and adduction–abduction motions, while the ankle joint had all 6 DOFs. Three translational and two rotational loads that had been calculated through OpenSim in the previous study^17^ were applied on the ankle joint in the model for simulating the ground reaction forces during the squatting motion. A time function of flexion angle was applied on the hip joint. The insertion locations of muscles and ligaments in Figure 3 were all determined from the OpenSim patient-specific musculoskeletal model. The time-varying muscle forces calculated through OpenSim in the previous study^17^ were also applied on the corresponding muscle springs in the axial direction in the FE model (Figure 3). Both muscle forces and ankle joint loads were simulated values without experimental validations. The simulations were performed by ensuring the residual loads on pelvis joint satisfy the criteria of simulation in OpenSim. The residual loads are non-physical supplementary loads for making the inverse kinematic result dynamically consistent with the measured ground reaction forces. The femur and tibia were replaced by ANSYS MPC184 rigid connection elements for reducing computational cost. The MPC184 rigid elements were defined, respectively, between the installation surface of the femoral component and the hip joint centre, and between the bottom surface of the tibial tray and the ankle joint centre.

Two tibiofemoral collateral ligaments, the lateral collateral ligament (LCL) and the medial collateral ligament (MCL), and two cruciate ligaments, the anterior cruciate (ACL) and the posterior cruciate (PCL), are shown in Figure 3 and were modelled as nonlinear springs with pretensions. The stiffness of these ligament bundles and the equations of their force–displacement curves were referenced from literature.^20,21^ The pre-strain values (−0.25 to 0.08) of the tibiofemoral collateral ligaments from the literature^20,21^ were applied initially; however, this resulted in a single-side-condylar lift-off from the tibial bearing due to insufficient contact forces between the femur and tibia. Therefore, all the tibiofemoral collateral ligaments pre-strains were changed to 0.1 to ensure tibiofemoral contact on both condyles during the squatting motion. The spring stiffnesses of the patellar ligaments were assigned nonlinear values based on the patellar ligament force–elongation relationships for men which had been experimentally measured by O’Brien et al.^22^

The component material properties (see Table 1) in the previous study^17^ were also used in this study except those for the tibial insert and patellar implant. These had previously been assigned to have the linear material properties of ultra-high-molecular-weight polyethylene (UHMWPE) to reduce computational cost. In this study, however, the stress–strain relationship of the UHMWPE^19,25^ was used to simulate its nonlinear elastic–plastic property. Contact pairs for the tibiofemoral and patellofemoral joints were defined using the ANSYS default contact setting (Augmented Lagrange). The element size of the contact surfaces of these two joints was 2 mm. The element size for the volume mesh was 4 mm. Mesh sensitivity was studied; as it resulted in less than 5% change in the predicted peak contact pressure, no further mesh refinement was needed.

**Table 1.** Material properties in the FE model.

	Elastic modulus (MPa)	Poisson’s ratio	Coefficient of friction	Density (kg/m^3^)
Tibial insert and patellar implant (UHMWPE)	550 (elastic region)	0.46	0.04	0.945 × 10^3^, [23]
Femoral component (cobalt–chrome alloy)	193,000	0.29	0.05	8.5 × 10^3^, [24]
Tibial tray (titanium alloy)	110,000	0.33		4.4 × 10^3^, [24]
Patellar bone (cortical bone)	17,580	0.3	0.8	1.85 × 10^3^, [19]

UHMWPE: ultra-high-molecular-weight polyethylene.

Laxity testing of the PFCLs shown in Figure 3(b) was conducted on the unresurfaced patella model with the assumed ligament stiffness of 2000 N/m and different pre-strain values of 0.1, 0.05, 0.01 and 0.001, respectively.

**Figure 4.**
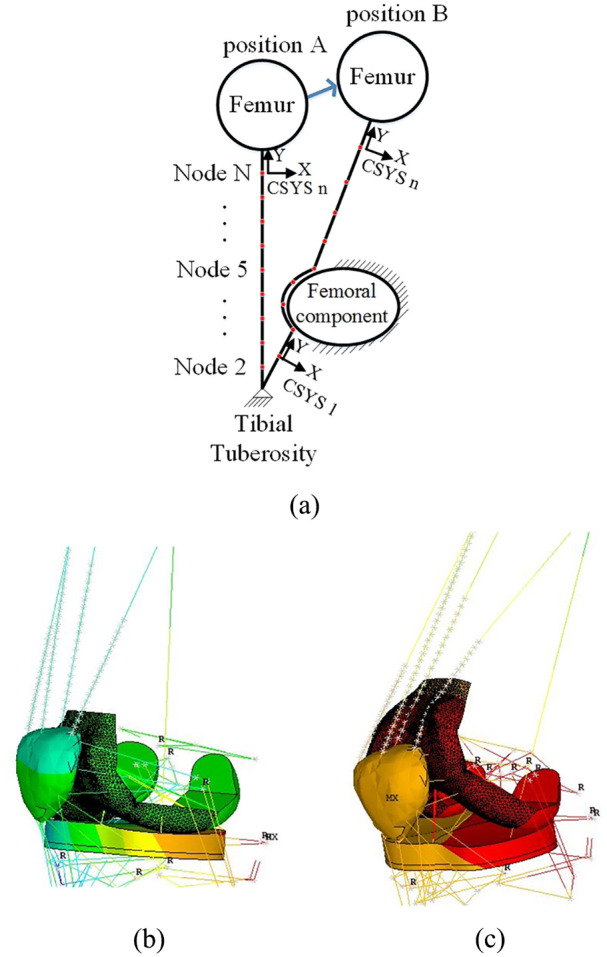
(a) Schematic of quadriceps muscle wrapping effect around femoral component: (b) low knee flexion position; (c) high knee flexion position.

To simulate the quadriceps muscle wrapping effect around the femoral component when the knee flexes, the four bundles of quadriceps shown in Figure 3(a) were split into N segments (element Combin14), each with spring stiffness equivalent to Ks*N (Ks: the stiffness of one bundle before discretization). Node-to-surface contact pairs were then defined using contact element CONTA175 in ANSYS Mechanical APDL from the (N + 1) nodes produced by the muscle discretization to the femoral component surface. For example, in Figure 4(a), it was assumed that the tibia with its bony tuberosity was fully fixed while the femur moved from position A to position B allowing the muscle springs to wrap around the femoral component. Please note that in Figure 4(a) the ellipse representing ‘femoral component’ is assumed to stay in the same position so as to make the discretised muscle deform by conforming to the shape of ‘femoral component’. In dynamic simulations, all 6 DOFs of the femoral component are free, and the femoral component movements are only constrained by the knee joint ligaments and the hip joint boundary conditions. Each spring element node was built in its local reference frame. In each local frame, the Y axis was defined as the axial direction of that spring element while the X axis was perpendicular to it in the sagittal plane. The Z axis pointed outwards from the X–Y plane. Once the spring node contacted the femoral component surface in Figure 4(a) during the course of its movement, each spring element between two spring nodes was then only allowed to deflect in the X–Y plane, with no random vibration in the Z direction. To ensure identical axial deflection of each muscle segment and to avoid any random vibrations in the Z direction, a set of coupling equations (equation (1)) were used to constrain DOFs of quadriceps muscle nodes in Y and Z directions. NY(i) and NZ(i) represent displacements of *i*th node in Y and Z directions

(1) $$\left\{ \begin{array}{c} \;\Delta_{\mathrm{NYi}}=\Delta_{\mathrm{NYi}-1}\\ \;\Delta_{\mathrm{NZi}}=\Delta_{\mathrm{NZi}-1}\\ \Delta_{\mathrm{NYi}}=\mathrm{NY}\left(i+1\right)-\mathrm{NY}\left(i\right)\\ \Delta_{\mathrm{NYi}-1}=\mathrm{NY}\left(i\right)-\mathrm{NY}\left(i-1\right)\\ \Delta_{\mathrm{NZi}}=\mathrm{NZ}\left(i+1\right)-\mathrm{NZ}\left(i\right)\\ \Delta_{\mathrm{NZi}-1}=\mathrm{NZ}\left(i\right)-\mathrm{NZ}\left(i-1\right) \end{array}\right.\;,i=1,2\;\sim \;N$$

The muscle wrapping effect is shown in Figure 4(b) and (c). With relative tibiofemoral movement, the bundles of quadriceps muscle successfully come into contact with the femoral component as the knee flexes from a small angle (Figure 4(b)) to a high angle (Figure 4(c)).

**Figure 5.**
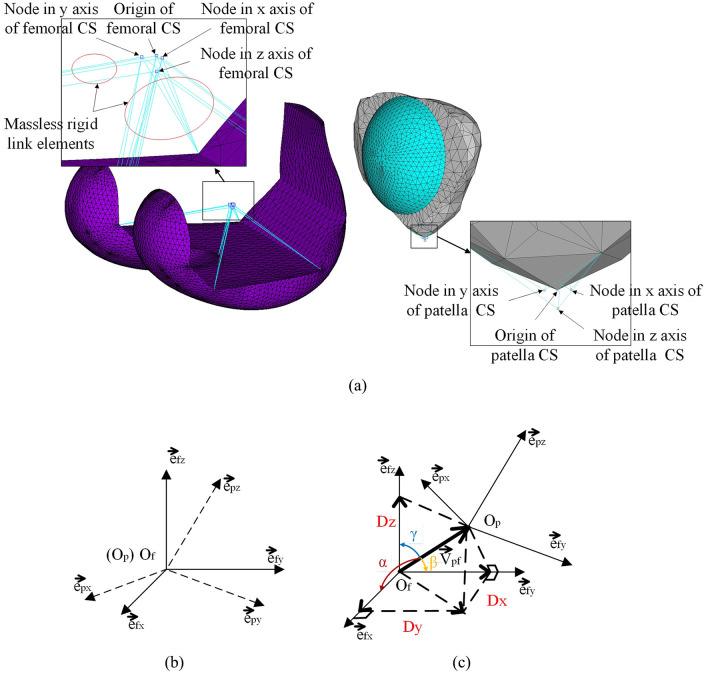
(a) Coordinate systems of femoral component and patella for tracking the patellofemoral joint relative motions: (b) rotation between two coordinate systems; (c) translation between two coordinate systems.

To study the relative motion between femur and patella, the patellofemoral motion was decomposed into rotational and translational motions. The rotations or Euler angles between the femoral and patellar coordinate systems (CSs) were calculated by applying a rotation matrix.^26^ As shown in each of the two detailed images in Figure 5(a), each CS was built through creating four nodes and then connecting them with five nodes on the femoral implant installation surface or three nodes on the patellar bone surface via massless rigid link elements. The rotations and translations were expressed both in the femoral CS and as a patellofemoral contact force (resultant of three vector forces along the femoral CS axes).

Since the two local reference frames moved with the two corresponding objects, the relative rigid body rotations could be obtained by solving the Euler angles between the two CSs shown in equations (2) and (3). In Figure 5(b), $e_{\mathrm{pi}},e_{\mathrm{fj}}(i,j=x,y,z)$ were the unit vectors in the patellar and femoral local frames, respectively. The femoral CS was regarded as the fixed CS, while the patellar CS was rotating relative to the femoral CS. The matrix in equation (2) was the transformation matrix of direction cosines^27^

(2) $$\begin{array}{l} \left(\begin{array}{c} e_{fx}\\ e_{fy}\\ e_{fz} \end{array}\right)=\left\{\begin{array}{ccc} Q_{xx} & Q_{yx} & Q_{zx}\\ Q_{xy} & Q_{yy} & Q_{zy}\\ Q_{xz} & Q_{yz} & Q_{zz} \end{array}\right\}\\ \left(\begin{array}{c} e_{tx}\\ e_{ty}\\ e_{tz} \end{array}\right),\;Q_{ij}=cos\left(e_{ti},e_{fj}\right)=e_{ti}\cdot e_{fj} \end{array}$$

(3) $$\left\{ \begin{array}{c} \theta_{x}=\mathrm{ta}n^{-1}\left(Q_{\mathrm{zy}}/Q_{\mathrm{zz}}\right)\\ \theta_{y}=\mathrm{ta}n^{-1}\left(-Q_{\mathrm{zx}}/\sqrt{Q_{\mathrm{zy}}^{2}+Q_{\mathrm{zz}}^{2}}\right)\\ \theta_{z}=\mathrm{ta}n^{-1}\left(Q_{\mathrm{yx}}/Q_{\mathrm{xx}}\right) \end{array}\right.$$

LaValle’s methods^26^ for solving equation (3) were used to obtain $\left(\theta_{x},\theta_{y},\theta_{z}\right)$. Rotation about the femoral x axis $\left(\theta_{x}\right)$ corresponded to the patellar flexion–extension rotation angle; rotation about the femoral y axis $\left(\theta_{y}\right)$ corresponded to the patellar external–internal rotation angle; and rotation around the femoral z axis $\left(\theta_{z}\right)$ corresponded to the patellar medial–lateral tilt angle.

The relative rigid displacements between femoral component and patella could be obtained from a simple triangulation calculation. In Figure 5(c), ${\vec{V}}_{\mathrm{pf}}$ is the distance vector between the patellar and femoral origins. α is the angle between ${\vec{V}}_{\mathrm{pf}}$ and $e_{\mathrm{fx}}$; *β* is the angle between ${\vec{V}}_{\mathrm{pf}}$ and $e_{\mathrm{fy}}$; and *γ* is the angle between ${\vec{V}}_{\mathrm{pf}}$ and $e_{\mathrm{fz}}$

(4) $$\left\{ \begin{array}{c} D_{x}=\left|{\vec{V}}_{\mathrm{pf}}\right|\cdot \cos\alpha ={\vec{V}}_{\mathrm{pf}}\cdot {\vec{e}}_{\mathrm{fx}}/\left|{\vec{e}}_{\mathrm{fx}}\right|\\ D_{y}=\left|{\vec{V}}_{\mathrm{pf}}\right|\cdot \cos\beta ={\vec{V}}_{\mathrm{pf}}\cdot {\vec{e}}_{\mathrm{fy}}/\left|{\vec{e}}_{\mathrm{fy}}\right|\\ D_{z}=\left|{\vec{V}}_{\mathrm{pf}}\right|\cdot \cos\gamma ={\vec{V}}_{\mathrm{pf}}\cdot {\vec{e}}_{\mathrm{fz}}/\left|{\vec{e}}_{\mathrm{fz}}\right| \end{array}\right.$$

$\left(D_{x},D_{y},D_{z}\right)$ are medial–lateral translation, anterior–posterior translation and inferior–superior translation of patella, respectively, relative to the femoral CS (represented by three axis nodes and one original node in Figure 5(a)).

### Initial conditions and solution setting for dynamic simulations

The FE model was not balanced initially for the ANSYS transient dynamic analysis under many time-varying loads. Some boundaries therefore needed to be constrained to ensure balance and convergence for the whole system. In this study, the tibial internal–external rotation DOF was fixed in the first 0.04 s along with mediolateral constraints on both sides of the patella. After 0.04 s, these constraints were removed allowing the patella and tibial components to move freely. However, they were still under the restraint of the collateral ligaments, retinaculum ligaments and PFCLs. Since pretension or initial force on the nonlinear spring element COMBIN39 is not allowed in ANSYS Mechanical APDL, the pretension load was converted into displacement load which was applied on those ligament springs in the first 0.01 s. After 0.01 s, the moving end of the spring element was connected to the ligament insertion point through the node-to-node contact settings for the rest of the simulation.

In terms of the control load steps in the ANSYS iteration solver, the maximum time-step was set at 0.01 s and the minimum 0.001 s. Automatic time stepping was also activated. These settings ensured that all the modes and responses of interest would be predicted.

## Results and discussions

### Contact forces on the patellofemoral joint

**Figure 6.**
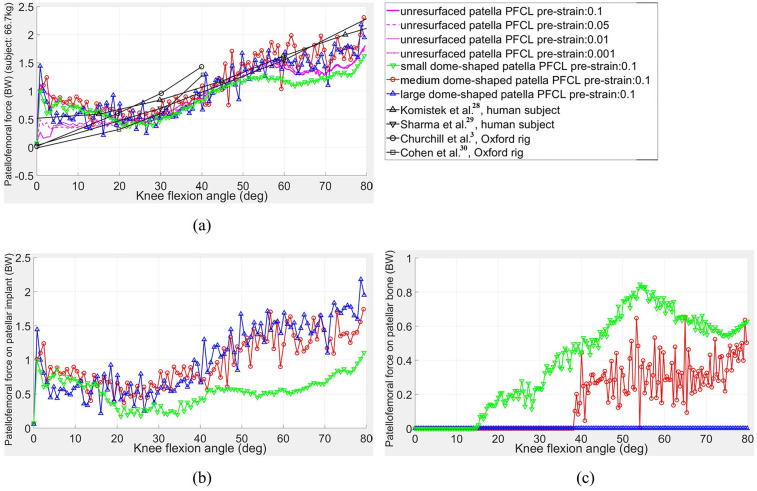
Patellofemoral contact forces (body weight (BW)) of unresurfaced patella model under different laxities of patellofemoral collateral ligaments (PFCLs) and three different-size patellar implants: (a) total patellofemoral contact forces and other study results; (b) patellofemoral contact forces on patellar implants; and (c) patellofemoral contact forces on patellar bones.

As can be seen in Figure 6(a), the patellofemoral contact forces of both unresurfaced and resurfaced models in this study were in good agreement with the referenced research results^3,28–30^ except at low knee flexion angles (up to about 20°) and very high flexion angles (approximately 60°–80°). The sudden load increase in the initial flexion was caused by releasing the constraints on the two sides of the patella. Because the patellofemoral and tibiofemoral joints were unstable under the ligament, musculotendon and joint reaction forces, the patella was only allowed to flex around and slide upwards or downwards along the femoral component in order to enable the simulation to converge quickly. The internal–external rotation of the ankle joint was also locked. At 0.04 s into the squatting simulation, the constraints on both patella and ankle joints were removed to allow the FE model to reach a new balance through its self-adjustment of the two contact pairs. These initial constraints could result in extra internal–external moment on the ankle joint and imbalance forces between the medial and lateral PFCLs; on removing the constraints, a sharp increased load was then produced on the given patellofemoral contact pair.

For the unresurfaced model, the differences in the early stage of simulations were mainly due to the laxities of the patellar collateral ligaments. The smaller the spring pre-strain, the smaller the reaction force produced between the femoral component and patella. To maintain the non-separation between patellar implant and femoral component during the squatting simulation, the PFCL pre-strain of 0.1 was applied on the three different-size patellar implant models. For the last 20° of knee flexion, the patellofemoral contact forces of both resurfaced and unresurfaced patella models were smaller than the results published by Komistek et al.^28^ and Sharma et al.^29^ This might be caused by the contact between the quadriceps muscle bundles and the femoral component; in our model, the quadriceps muscle bundles were allowed to wrap around the distal femur whereas the aforementioned two researchers used a cylindrical joint mechanism in their simulations. In general, the trend of the simulated patellofemoral forces agreed well with the existing research results.^3,28–30^ The small dome-shaped patella model resulted in smaller load than the other two dome-shaped models due to its reduced moment arm to the patellofemoral joint centre.

The small dome-shaped patella model was found to have smaller contact forces between the femoral component and patellar implant as shown in Figure 6(b), which was mainly caused by the contact between the patellar bone and femoral component from 15° knee flexion (see Figure 6(c)). The load fluctuation in the small dome-shape patella model was also smaller than the other two patellar implant models. In Figure 6(c), bone-component contact also occurred in the medium implant model though at a higher flexion angle (38°) than with the small implant. There was still some bone uncovered on the two sides and superior areas of the patella in the medium dome-shaped patella model as shown in Figure 2. In order to avoid all bone-component contact, a large dome-shaped patella had been created by continuing to scale up the dome-shaped patellar implant. Although no bone-component contact then occurred as shown in Figure 8(c), the large dome-shaped patellar implant extended beyond the superior area of the resected patellar bone surface as shown in Figure 2, and this might lead it to contact with the quadriceps muscle during the knee flexion.

### Relative motions of the patellofemoral joint

**Figure 7.**
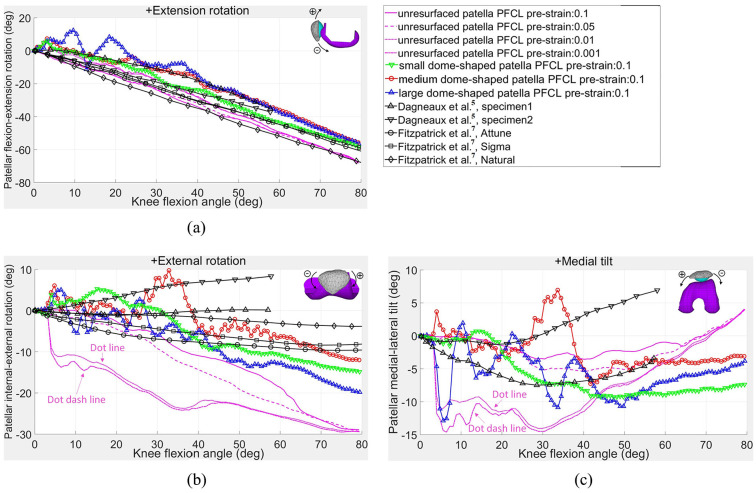
Relative motions of patella over femoral component: (a) extension–flexion rotation; (b) external–internal rotation; and (c) medial–lateral tilt.

**Figure 8.**
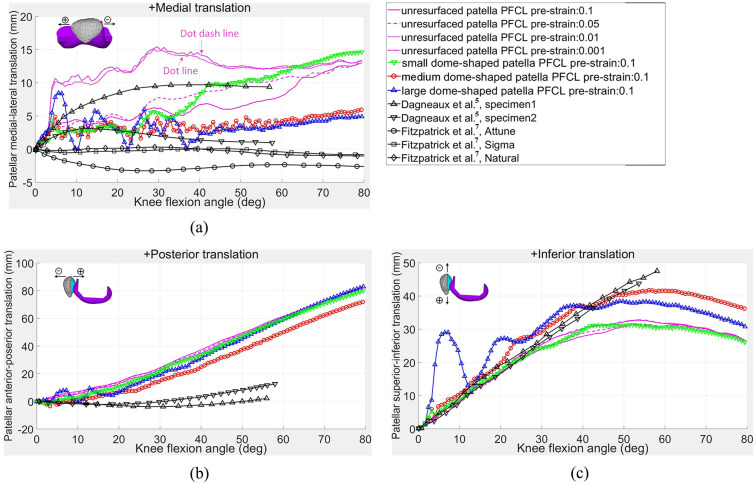
Relative motions of patella over femoral component: (a) medial–lateral translation; (b) anterior–posterior translation; and (c) inferior–superior translation.

The relative motions between the patella and femoral component are presented in Figures 7 and 8. In Figure 7(a), the patellar flexions in the resurfaced and unresurfaced models were consistent with the results simulated by Fitzpatrick and Rullkoetter^7^ and those measured by Dagneaux et al.^5^ Regarding the external–internal rotation of the patella, as shown in Figure 7(b), the resurfaced patella under the boundary conditions of this study first rotated towards the lateral side of knee joint till the knee flexed to 20°, and then internally rotated similar to the results obtained by Fitzpatrick and Rullkoetter.^7^ This was mainly due to the PFCL pre-strains that were assumed values rather than practically measured ones. Once the lateral side PFCLs produced larger tension forces than the medial side, the patella would inevitably rotate externally. By contrast, the unresurfaced patella under different PFCL pre-strains only rotated linearly towards the medial side of knee joint.

The trends and magnitudes of the unresurfaced patella in medial tilt rotation shown in Figure 7(c) and medial translation in Figure 8(a) matched well with the measured results from Dagneaux et al.,^5^ except that sharp increases at 4° knee flexion were observed in the magnitude of the two patellar motions when the pre-strain of the PFCLs was less than 0.01. This was mainly caused by the pretension forces in the PFCLs being insufficient to resist the medial patellofemoral force, which caused the patella to suddenly slide towards the medial side of knee joint. The same situation appeared in the patellar external rotation (Figure 7(b)) as well.

The differences between the resurfaced and unresurfaced patellae in the patellar medial tilt and internal rotation might be due to the different bearing surfaces between the button-shaped implant and saddle-shaped natural patella. In addition, the patellar internal rotations in both resurfaced and unresurfaced patellae were different to those of test specimens measured by Dagneaux et al.,^5^ which might be due to the differences in the loading boundary conditions and the shapes of the femoral trochlear groove. It should be pointed out that the patellar motion results in the literature^5,7^ were only used as a reference for preliminary model validation to provide a general range and pattern of patellar motion. Further experiment based on the simulation hypothesis conditions is needed for further validating the models and calculated results.

The patellar posterior translation motion shown in Figure 8(b) was almost linearly proportional to the knee flexion angle, whereas there was only a slight posterior translation in Dagneaux’s results.^5^ In Figure 8(c), the inferior translations of both resurfaced and unresurfaced patellae were in good agreement with Dagneaux’s results^5^ in the first 30° and 50° of knee flexion. Thereafter, the patellar inferior translation in this study gradually reached 31 or 41 mm at 55° knee flexion. The differences between this study and the Dagneaux study^5^ in the patellar posterior and inferior translations might be due to the smaller load applied on the subject ankle joint in the Dagneaux study.^5^ A differently shaped trochlear groove that guides the sliding of the patella on the femur could also affect the patellar motion trajectory.

The laxity of the PFCLs was important in the dynamic simulations of the knee joint. If the initial strain in the PFCLs was too small to provide enough pretension on the patellofemoral joint, the relative motions between the patella and femoral component would show malposition or separation of the patella from the femoral component during the dynamic simulations. In this study, due to the lack of relevant data about the mechanical properties of the PFCLs, the ligament stiffness and initial strain of the PFCLs were assumed based on knowledge about the knee collateral ligaments. The results of the patellofemoral and tibiofemoral joints could only show the trends in their responses to the design parameters through this nonlinear dynamic knee simulation model.

### Contact stresses of the patellofemoral joint

**Figure 9.**
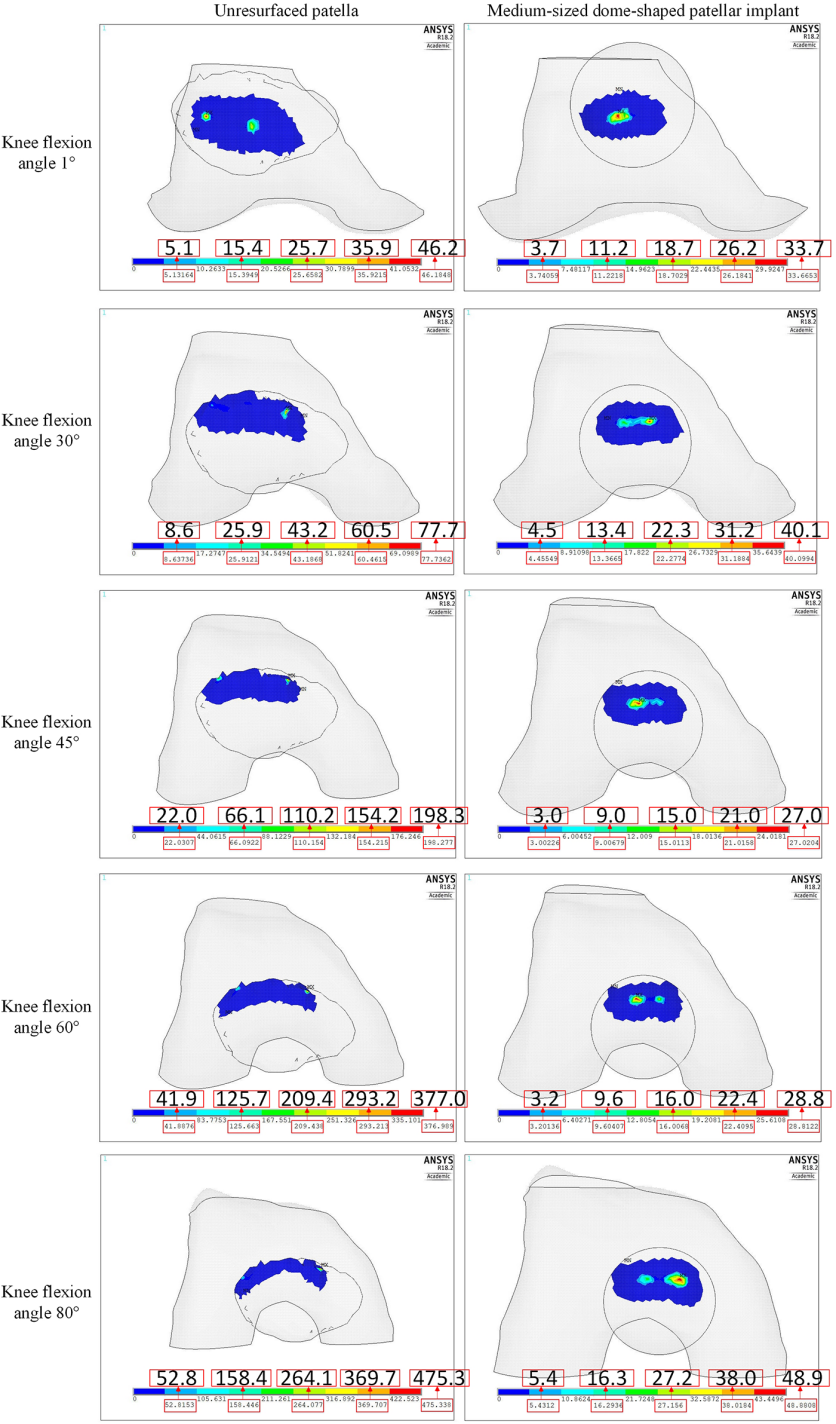
Patellofemoral normal contact stresses (MPa) of CTKIs with unresurfaced and resurfaced patellae.

The patellofemoral contact stresses are presented at flexion angles of 1°, 30°, 45°, 60° and 80° in Figure 9 for the unresurfaced patella and the resurfaced patella with the medium-sized dome-shaped implant. During knee flexion, the contact location on the resurfaced patella shifted from the centre of the implant button to the superior right area along with medial translation of the patella, while the contact locations on the unresurfaced patella changed from the centre to the superior side due to the restraint by the saddle-shaped articular surface of the patella. The reduced contact area of the unresurfaced patella with its superior-moving motion may help explain its linear rotation towards the medial side of knee joint in Figure 7(b) due to its reduced pivot effect. Excessively high contact stresses observed on the unresurfaced patella were due to the incongruent surfaces of the patellofemoral joint and the unsmooth patellar articular surface. The unresurfaced patella was built from CT images using three-dimensional (3D) Slicer and consisted of several discontinuous small surfaces; localised stresses then resulted. The contact area on the medium-sized dome-shaped patellar implant gradually changed from single contact area at 1° knee flexion to dual contact areas at 60° knee flexion.

**Figure 10.**
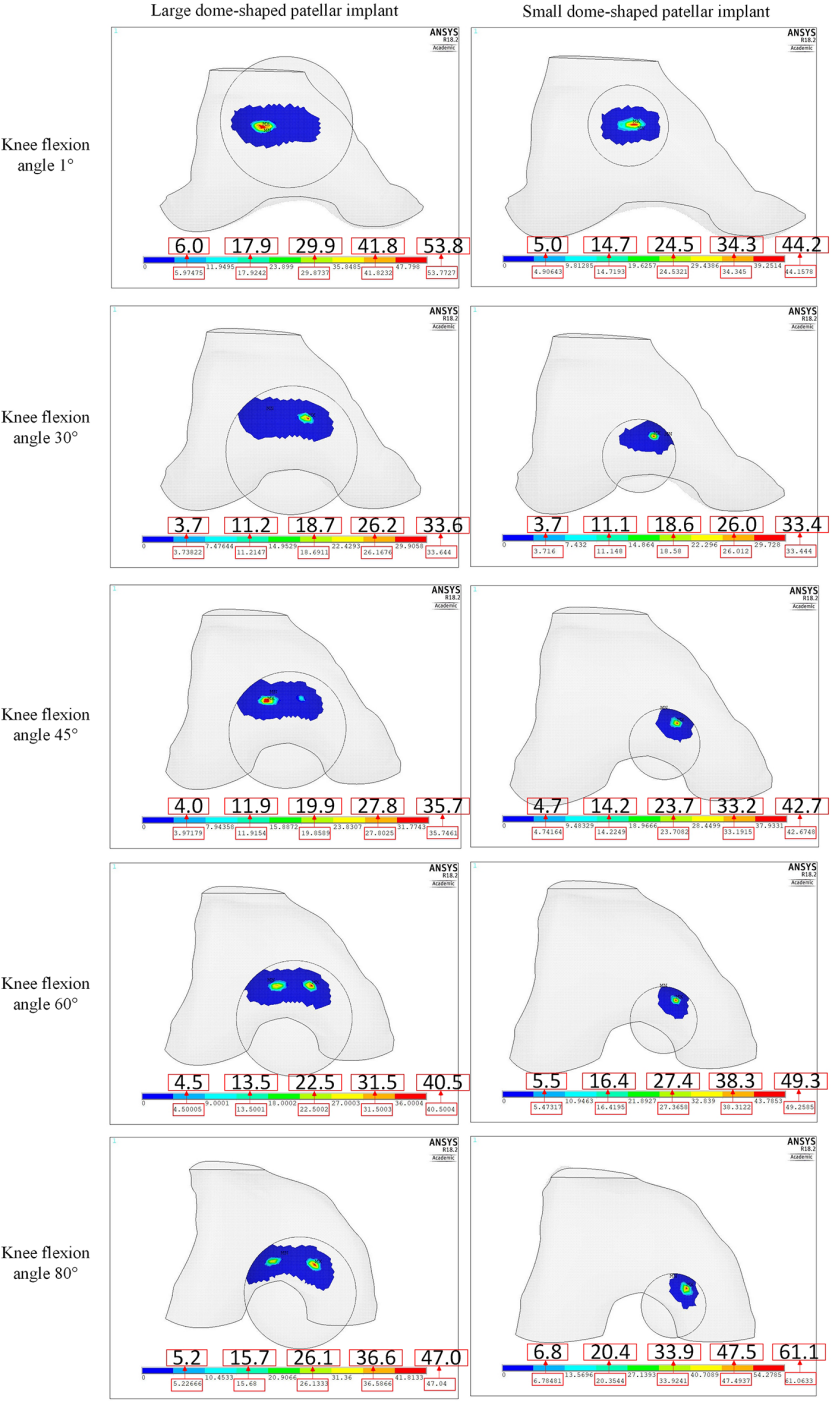
Patellofemoral normal contact stresses (MPa) of CTKIs with large and small dome-shaped patellar implants.

The contact stresses of the large and small dome-shaped patellar buttons are shown in Figure 10. The contact area on the large dome-shaped patellar implant changed from a single contact area at 1° knee flexion to dual contact areas at 45° knee flexion. In contrast, the small dome-shaped patellar implant always had only a single contact area during knee flexion. Therefore, the contact stress was larger on the small dome-shaped patellar implants than those on the two larger dome-shaped patellar implants above 45° knee flexion. The higher medial translation of the small dome-shaped patellar implant in comparison to that of the large patellar implant (Figure 10) is consistent with the results shown in Figure 8(a) and might be related to the smaller restriction effect of the single contact area on the small dome-shaped patellar implant. The big difference between the medium dome-shaped patellar implant model in Figure 9 and the large patellar implant model in Figure 10 in some contact stresses at 45° and 60° knee flexions might be due to the instantaneous difference of implant-component contact force that was shown in Figure 6(b) apart from the difference of contact area size.

The fluctuation of result curves in Figures 6–8 was mainly caused by the solver algorithm adopted in ANSYS. The penalty method was used to detect and control the contact between two contacting objects. A certain amount of penetration depth of object A into object B is allowed but has to be within a tolerance. If the depth is exceeded, the object A will return to a position in relation to the object B until the convergence criteria is met. Apart from that, since we used transient analysis in ANSYS, the force balance is another convergence criterion that has to be met. The difference between system external forces and internal forces (the tibiofemoral and patellofemoral contact forces) needs to be within a tolerance. In addition to the fact that the patellofemoral joint having asymmetric structure and loading (four time-varying forces on the superior part of patella and three passive ligament springs on the inferior), it turned out to be bouncing effect between patella and femoral component in terms of their reaction forces and relative displacement.

### Limitations of study

There are limitations in the modelling of the knee joint. First, the cartilage on the unresurfaced patella was not considered, and it might affect the motions and loads of the patellofemoral joint. Second, the patellar articular surface was modelled as several small, irregular and unsmooth surfaces from the medical image processing software, 3D Slicer, which resulted in the excessive patellofemoral contact stresses. Smoothing the patellar surface may help reduce the contact stress. Third, the initial location of the patella in relation to the femur was based on the CT images, but adjusted due to the different sizes of patellar implant and different shapes between unresurfaced and resurfaced patellae. This could lead to different results, especially the patellar posterior and inferior translations. Nevertheless, the simulations can generally predict the trend of patellofemoral joint motion and contact locations to allow comparisons of different designs of patellar implant.

## Conclusion

In this article, dynamic FE simulations driven by patient-specific muscle forces and ankle joint forces were successfully used to investigate the influence of the unresurfaced patella and three dome-shaped patellar implants on the CTKI patellofemoral joint contact forces, relative motions and contact stresses. Differences in the motions of patellar internal rotation and medial tilt were found between the resurfaced and unresurfaced patellar implants. The size of the dome-shaped patellar implant was found to affect the motion and loading of the patellofemoral joint. When the size of the patellar implant was not sufficient to cover the resected patella, the exposed patellar bone could contact the femoral component during knee flexion, which would cause discomfort or pain in the knee joint. The dome-shaped patellar implants could also change the patellar movement relative to femur compared to natural unresurfaced patella. Either sustaining patient’s own patellar articular surface or designing a customised one may help patient’s knee move more naturally. In general, the computer models in this article can predict the trends in the motion and forces in the patellofemoral joint for different designs of patellar button implant under physiological muscle forces and ankle joint loads.
